# Obesity, Weight Gain, and Fluid Overload in Peritoneal Dialysis

**DOI:** 10.3389/fneph.2022.880097

**Published:** 2022-06-28

**Authors:** Jack Kit-Chung Ng, Win Hlaing Than, Cheuk Chun Szeto

**Affiliations:** ^1^ Carol & Richard Yu Peritoneal Dialysis Research Centre, Department of Medicine & Therapeutics, Prince of Wales Hospital, The Chinese University of Hong Kong, Shatin, Hong Kong SAR, China; ^2^ Li Ka Shing Institute of Health Sciences (LiHS), Faculty of Medicine, The Chinese University of Hong Kong, Shatin, Hong Kong SAR, China

**Keywords:** chronic kidney disease, atherosclerosis, metabolic syndrome, inflammation, cardiovascular disease

## Abstract

Obesity is a global epidemic that has a complicated pathogenesis as well as impact on the outcome of peritoneal dialysis (PD) patients. In this review, the prevalence of obesity in incident PD patients as well as the phenomenon of new-onset glucose intolerance after PD will be reviewed. Published literature on the effect of obesity on the survival and incidence of cardiovascular disease in PD patients will be discussed. Particular emphasis would be put on literature that compared the impact of obesity on the outcome of hemodialysis and PD, and the confounding effect of dialysis adequacy. Next, the complex concept of obesity and its relevance for PD will be explored. The focus would be put on the methods of assessment and clinical relevance of central versus general obesity, as well as visceral versus subcutaneous adipose tissue. The relation between obesity and systemic inflammation, as well as the biological role of several selected adipokines will be reviewed. The confounding effects of metabolic syndrome and insulin resistance will be discussed, followed by the prevalence and prognostic impact of weight gain during the first few years of PD. The differences between weight gain due to fluid overload and accumulation of adipose tissue will be discussed, followed by the current literature on the change in body composition after patients are put on chronic PD. The methods of body composition will be reviewed, and the clinical relevance of individual body component (fluid, fat, muscle, and bone) will be discussed. The review will conclude by highlighting current gaps of knowledge and further research directions in this area.

## Introduction

Peritoneal dialysis (PD) is widely used for providing home-based dialysis (1, 2). Traditionally, PD-related peritonitis has long been the Achilles heel of PD and the major cause of technique failure (3, 4). With the advances in connection system and standardization of treatment protocol, the focus of improving the longevity of PD patients has shifted to metabolic complications and cardiovascular diseases (5, 6). In addition to the high prevalence of traditional cardiovascular risk factors, it is increasingly recognized in recent years that non-traditional risk factors play important roles in the pathogenesis of cardiovascular disease in PD patients (7, 8). Specific uremic toxins, anemia, disturbances in divalent ion metabolism, sympathetic nerve over-activity, gut dysmotility, circulating bacterial metabolites and fragments, and various treatment-related factors may all contribute to the pathogenesis of cardiovascular disease in CKD (9, 10). More recently, obesity has emerged as the key modifiable risk factor that bridges the traditional and non-traditional pathogenic pathways of cardiovascular disease.

## Definition of Obesity

Obesity is most commonly defined by body mass index (BMI). The World Health Organization (WHO) considers a BMI between 20 and 25 kg/m^2^ as normal weight, a BMI between 25 and 30 kg/m^2^ as overweight, and a BMI of >30 kg/m^2^ as obese (11). However, the Asian population have a higher body fat content for the same BMI than the western population (12), and the International Obesity Task Force recommended the lower cut-offs of BMI ≥23 kg/m^2^ for overweight, and ≥25.0 kg/m^2^ for obese for Asian people (13). To complicate the matter, BMI is a poor estimate of fat mass distribution in CKD (14). Although waist-hip ratio (WHR) and skin fold thickness are superior to BMI for the correct classification of obesity in CKD (15), skin fold thickness is not readily available in most centers, and WHR may not be a valid estimate in PD patients (16).

## Epidemiology of Obesity

Following the trend in the general population (17–21), obesity is increasingly common among incident PD patients. In a retrospective study of 1681 incident adult PD patients from a single center, Than et al. (22) reported that 37.7% were obese or overweight at the initiation of PD over a 25 years period of observation. In this study, the prevalence of obesity or overweight at the initiation of PD increased from 21.9% before 2000 to 47.3% after 2015 (22). Notably, the absolute increase in the prevalence of obesity or overweight was more pronounced in diabetic patients (from 33.7% to 59.6%) than non-diabetic ones (from 13.2% to 32.3%), although the relative increase was actually more marked in the non-diabetic group (22). However, the prevalence of obesity as well as the magnitude of its increase was slightly lower among PD patients than that observed in the general population (21), which is probably the result of the common coexistence of protein-energy malnutrition in patients with advanced CKD (23, 24).

## Assessment of Obesity and Body Fat Content

Traditionally, body mass index (BMI) is the most commonly used marker for obesity (17–21). BMI is simple and easily understood by patients. However, BMI tends to over-diagnose obesity in tall patients (12567), and does not distinguish the cause of a high body weight, which may be due to the increase in muscle mass or fluid retention (25). In recent years, a number of specific tests, notably multi-frequency bioimpedance spectroscopy and dual-energy X-ray absorptiometry (DXA) scan, have been developed for the measurement of individual body compartments (26). Multi-frequency bioimpedance spectroscopy measures the resistance and reactance of the body under the flow of electrical current and provides a reproducible and non-invasive method to determine the adipose tissue mass and volume of overhydration in the body (27). Since the equipment is simple and the method is suitable for frequent repeated measurements, the technique has been extensively used in hemodialysis as well as PD patients (28–30), but the technique is more commonly used for the monitoring of body fluid status rather than fat content of dialysis patients. DXA scan uses two X-ray beams with different energy levels to perform spectral imaging that allows the measurement of bone mineral density as well as body fat and muscle distribution (31). However, the equipment is expensive and the application in the dialysis population is less well reported.

## Weight Gain During PD

Not only that obesity is common in incident PD patients, a large proportion of PD patients have substantial weight gain after the initiation of PD (32–34). In a study of 444 consecutive incident PD patients, Choy et al. (35) found that the mean weight gain after one year of PD was 1.34 kg, and nearly 25% patients had weight gain over 3 kg. Nonetheless, previous studies that compared the magnitude of the weight gain after the initiation of dialysis showed either no significant difference in the change in body weight after started on PD or hemodialysis, or actually a slightly higher probability of substantial weight gain after started on hemodialysis (36, 37). In the study of Choy et al. (35), there were no significant correlations between body weight gain and glucose load or peritoneal transport parameters, but patients without any peritonitis episodes during the first year of PD had significantly more weight gain than patients who experienced peritonitis during that time (35). Taken together, these data indicate that improvement in uremia and general health is the more important cause of weight gain, while glucose absorption from the PD solution plays only a minor role. Consistent with this notion, weight gain during the first year of PD in this study was not associated with adverse clinical outcome in the subsequent follow up, while weight loss during the first year of PD predicted poor patient survival (35). Similarly, analysis of 1911 adult incident PD patients recruited from 114 dialysis centers that participated in the Brazilian Peritoneal Dialysis Multicenter Cohort Study, Fernandes et al. (38) found that weight gain during the first year of PD was not associated with a higher subsequent mortality. In contrast, in an observational study of 148 incident PD patients, Kim et al. (39) noted that excess weight gain during the first year of PD was closely linked to systemic inflammation, diabetes and rapid decline in residual renal function, although the effect on subsequent mortality was not significant. In another prospective study of 109 incident PD patients, Castro et al. (40) found that 61% had an increase in waist circumference after 6 months of PD, and a significant increase in waist circumference was only observed among patients who died in the subsequent 4 years of follow up, suggesting that weight gain after PD may not be an entirely benign phenomenon.

With a longer duration of observation, our recent analysis of 954 consecutive incident PD patients found that the average weight gain was 1.2 kg after the first 2 years of PD, and the magnitude of weight gain after PD increased progressively over the past 25 years (41). In this analysis, there was a significant interaction between baseline body mass index (BMI) and subsequent weight change on technique survival but not on patient survival. For the patients with baseline BMI <23 kg/m2, weight gain ≥3 kg was associated with a better 5-year technique survival, while for patients with baseline BMI >25 kg/m2, weight gain ≥3 kg was associated with a trend of worse technique survival (41). On the other hand, weight gain ≥3kg was associated with a worse subsequent patient survival rate irrespective to the baseline BMI (41). Taken together, available literature does not show a consistent effect of weight gain after the initiation of PD, prognostic impact probably depends on the baseline nutritional status of the patient.

## The Obesity Paradox in Advanced CKD

In the general population, obesity is a well-known risk factor of metabolic diseases (e.g. type 2 diabetes mellitus, non-alcoholic fatty liver disease) and cardiovascular diseases, as well as Alzheimer disease, depression, osteoarthritis, and several types of cancer (17, 42, 43). In addition, obesity is also well reported to contribute to the pathogenesis and progression of CKD (44, 45). In CKD patients, however, the role of obesity as a cardiovascular risk factor is less well studied. In a cross-sectional study of 1740 patients with stage 3 CKD, the degree of central obesity, as defined by the waist-hip ratio, had a strong and independent correlation with arterial pulse wave velocity (46). In another observational study of 1669 patients with stage 2 to 4 CKD followed for an average of 9.3 years, Elsayed et al. (47) found that waist-hip ratio, but not BMI, was associated with cardiovascular events, indicating that central obesity is more important than BMI as a cardiovascular risk factor.

Contrary to the observations in the general population, obesity is often reported to be associated with a better outcome in patients receiving kidney replacement therapy (KRT), which is mostly chronic hemodialysis in the published literature. In a review of the US Renal Data System data in 418,055 hemodialysis patients, Johansen et al. (48) found that high BMI was associated with increased patient survival, including the subgroup with extremely high BMI. In this study, high BMI was also associated with a reduced risk of hospitalization and a lower rate of mortality in all mortality categories (48). Notably, different methods for measuring adiposity, including the Benn index and estimated fat mass, yielded similar results, and adjustments for lean body mass did not affect the findings (48). Similarly, a systemic review of 10 studies with over 1 million patients showed that hemodialysis patients with higher body weight or BMI were associated with a lower all-cause mortality and cardiovascular mortality, and the benefit was consistent across all ethnic groups (24). In another meta-analysis of 22 studies on hemodialysis patients, the relationship between BMI and mortality was linear (49). In this analysis, 1 kg/m^2^ increase in BMI was associated with a 3% reduction in all-cause mortality, and 4% reduction in cardiovascular mortality (49).

## Impact of Obesity on the Outcome of PD Patients

Published data on the relation between obesity and the outcome of PD patients are limited and showed conflicting results (**Table 1**). Snyder et al. (50) and Ramkumar et al. (51) reported that overweight and obese PD patients have better survival rates than those with lower BMI, while McDonald et al. (52) noted that obesity was associated with higher risks of death and technique failure. Other early studies did not find any association between obesity and the outcome of PD patients (53–55). In a study of 280 PD patients, Liao et al. (56) found that metabolic syndrome was a significant predictor of cardiovascular events in non-diabetic patients. However, when the 5 components of metabolic syndrome were separately analyzed, only hypertriglyceridemia, low high-density lipoprotein levels, and hyperglycemia predicted adverse cardiovascular outcomes, while central obesity was not a significant prognostic indicator (56). The conflicting results may be explained because BMI may not be a marker of obesity but that of nutritional status. Incident PD patients with high BMI may have better survival if they have a high skeletal muscle mass rather than adipose tissue mass (51). In a systematic review, Ahmadi et al. (57) concluded that the relation between BMI and mortality was linear during the first year of PD. In this analysis, being underweight was associated with higher mortality, and being overweight or obese, as defined by BMI, was associated with lower mortality. The relation, however, became an insignificant J-shape one after 2 years on PD, and both underweight and obesity seemed to increase the mortality risk (57). The same J-shape relationship between BMI and mortality was also noted in another meta-analysis that focused on Asian PD patients (58). It is important to note that all these studies focused on all-cause mortality, and there are few published data on the association between obesity and cardiovascular mortality in PD patients (49), although a high BMI was protective for all-cause mortality as well as cardiovascular mortality in the hemodialysis populations (49). Although obese PD patients had higher risk for complications than non-obese PD patients, their survival was similar to matched HD patients (59), indicating that obesity is not a contraindication for PD.

**Table 1:** Relation between obesity and the outcome of PD patients.

Author	No. of case	fFllow up (year)	Findings
Snyder (50)	41,197	3	obese patients have better survival: adjusted HR of mortality 0.89 (p < 0.05), 0.99, and 1.00 for the obese in the first, second, and third year, respectively
Ramkumar (51)	10,140	1.5	new PD patients with high BMI/skeletal muscle mass have best survival: HR of all-cause mortality 0.90 (95%CI 0.83-0.97); HR of CV mortality 0.88 (95%CI 0.79-0.97)
McDonald (52)	9679	2	obesity associated with death (HR 1.36; 95%CI 1.14-1.54; p < 0.05) and technique failure (HR 1.17; 95%CI 1.07-1.26; p < 0.01)
Abbott (53)	1662	5	BMI over 30 not associated with survival risk or advantage (adjusted HR 0.99; 95%CI, 0.86-1.15; p=0.89)
Stack (54)	17,419	1	higher mortality for BMI < 20.9 (RR 1.20, 95%CI 1.00-1.43 for diabetic; RR 1.39, 95%CI 1.19-1.64 for nondiabetic); no difference in mortality between BMI quintiles 20.9-23.5, 23.5-26.1, 26.1-30.0, and >30.0
De Mutsert (55)	688	5	new PD patients with BMI >30 have similar survival with those with BMI 18.5-25: HR 0.8 (95%CI 0.5-1.3)
Liao (56)	280	4	metabolic syndrome was independently associated with increased risk for CV death (HR 13.3) and fatal or non-fatal CV events (HR 10.5); obesity (BMI >25) did not affect all cause mortality (HR 1.45; 95%CI 0.57-3.70; p =0.43), CV death (HR 2.02; 95%CI 0.59-6.88; p =0.26), and fatal or non-fatal CV events (HR 1.76; 95%CI 0.69-4.52; p=0.24)
Ahmadi (57)	156,562 (9 studies)	5	first year of PD: BMI 25-30 was associated with lower mortality than BMI 18-25 (HR 0.83, 95%CI 0.79-0.89) 3 to 5 years after PD: insignificant J-shape relationship with BMI 18-25 as the reference, i.e. BMI >30: HR 1.07 (95%CI 0.93-1.23); BMI < 18: HR 1.17 (95%CI 0.95-1.44)
Liu (58)	3610 (7 studies)	variable	J-shape relationship: obese group (BMI 25-29.9) associated with higher risk of all-cause mortality (HR 1.46; 95%CI 1.07-1.98; p = 0.02) and CV mortality (HR 2.01; 95%CI 1.14-3.54; p = 0.02); underweight group (BMI < 18.5) also had higher all-cause mortality (HR 2.11; 95%CI 1.46-3.07; p < 0.001).
Obi (59)	15,573	4	obese PD patients had higher risk for hospitalization for peritonitis and technique failure (i.e. need of transfer to hemodialysis) (P for trend < 0.001 for both analyses)

PD, peritoneal dialysis; BMI, body mass index (in kg/m^2^); CV, cardiovascular; HR, hazard ratio; CI, confidence interval.

There are recent data to further show that the prognostic impact of obesity is affected by the coexistence of frailty. In a study of 267 prevalent Chinese PD patients, Chan et al. (60) noted that frail patients had a higher waist-hip ratio (an indicator of central obesity) but not BMI. Although waist-hip ratio did not predict patient survival in this study, there was a significant interaction between waist-hip ratio and frailty on patient survival and cardiovascular survival (60). For patients without frailty, the two-year cardiovascular survival was significantly better for those with a high waist-hip ratio (91.3% versus 74.4%), and they had fewer hospital admission for cardiovascular disease in 2 years, while waist-hip ratio did not predict the cardiovascular survival or need of hospital admission for cardiovascular disease in frail patients (60). The result of this study seems to indicate that there is a protective role of obesity in non-frail PD patients but not the frail ones (60).

## Impact on PD Catheter Insertion and Complications

Obesity is well reported to be associated with PD catheter-related complications, which include catheter malfunction and various mechanical complications. Common sense and clinical experience suggest that obese patients have more technical difficulty with PD catheter insertion because of the deeper operating field, but published literature in this area is limited (61, 62). Visceral adiposity is associated with an increased volume of omentum, which theoretically increases the risk of catheter malfunction due to omental wrap. Again, there are no good data to support this notion, and results are conflicting from published study regarding the benefits of prophylactic omentectomy or omentopexy at the time of catheter insertion (61). A study that compared PD catheter insertion by mini-laparotomy, simple laparoscopy, or advanced laparoscopic techniques (with rectus sheath tunneling, selective omentopexy, and adhesiolysis) found no difference in the rate of catheter malfunction rates in the entire study population as well as the subgroup of obese patients (63). An observational study suggests that extended catheters have satisfactory survival compared with conventional catheters (64), but extended catheters are not generally available in many countries.

## Impact on Dialysis Adequacy

An important reason that obesity may affect the outcome of PD patients is the impact of body size on dialysis adequacy. In essence, the capacity of the peritoneum as a dialysis membrane is intrinsically limited by its relatively low permeability (as compared to synthetic membrane of hemodialyzers) and the maximal time allowed for dialysis each day (i.e. 24 hours). In the scenario of continuous ambulatory peritoneal dialysis (CAPD), the problem is aggravated by a generally fixed dialysis regimen of 3 or 4 daily exchanges, or 6 to 10 L/day for the usual 2 to 2.5 L exchange volume (65). The surface area of peritoneal membrane is similar to body surface area, which is proportional to the square root of body weight (66), implying that obese patients have a proportionally “smaller peritoneal dialyzer” (i.e. lower area of peritoneal membrane for dialysis). Assuming complete equilibration of urea in the PD effluent, a CAPD regimen of four 2-L exchanges per day in a patient with body weight 50 kg (i.e. total body water 30 L) would achieve a weekly peritoneal Kt/V 1.87 (67). The same CAPD regimen in a patient with body weight 70 kg would achieve a weekly peritoneal Kt/V 1.33, which would increase to 1.67 if the regimen is adjusted to four 2.5-L exchanges (67). For a 100 kg patients, a regimen of four 3-L exchanges (which is the maximal tolerable dwell volume without respiratory compromise) would only have a peritoneal Kt/V 1.4.

Clinical observations are in line with the above-mentioned theoretical considerations. Obesity generally has little adverse effect on the all-cause mortality of incident PD patients (49, 57). In a meta-analysis of 9 studies, Ahmadi et al. (57) found that being overweight or obese was associated with lower 1-year mortality and no significant association with 2-, and 3- to 5-year mortalities. Given the above discussion on the limitations of PD in providing adequate solute clearance for patients with a higher body weight, the gradual loss of survival benefit for PD in obese patients could possibly due to the loss of residual renal function. More importantly, the probability of conversion to hemodialysis increases substantially with the BMI. In an observational study of 15,573 incident PD patients from 2007 to 2011, Obi et al. (59) found that obese patients had faster declines in residual kidney function and consistently achieved lower total Kt/V over time despite greater increases in dialysis Kt/V than non-obese patients. More importantly, higher BMI was significantly associated with shorter time to transfer to hemodialysis (59), suggesting that dialysis adequacy and small solute clearance are the major concern of obese PD patients.

## Obesity and Inflammation

The major mechanism that explains the potential link between obesity and the adverse clinical outcome in the general population is systemic inflammation (68), but the data dedicated to the PD population in this area are scarce. In essence, obesity should be considered as a chronic inflammatory state because adipocytes secrete a panel of peptides mediators, commonly referred to as adipokines (69), that play important roles in the pathogenesis of insulin resistance, endothelial dysfunction, and cardiovascular disease (68, 70). Traditional cytokines released by the adipose tissue include tumor necrosis factor-α (TNF-α), interleukin-6 (IL-6), plasminogen activator inhibitor (PAI-1), monocyte chemotactic protein-1 (MCP-1), and macrophage migration inhibitory factor (MIF) (71–74). They play important roles in the local regulation of adipose tissue metabolism as well as triggering a pro-inflammatory state in distant sites (75–79).

Adipocytes also secrete a panel of mediators that are usually not produced by other cell types. Notable candidates include leptin, adiponectin, resistin, omentin-1, and vaspin. Leptin, the best known adipokine, affects nitric oxide production and activates the sympathetic system (80, 81). Increases in serum leptin levels during PD are associated with inflammation and a decrease in lean body mass (82). Adiponectin is another major adipokine that is involved in regulating glucose levels and fatty acid breakdown (83). Resistin was first described to be involved in the pathogenesis of obesity-associated insulin resistance in mice (84). Omentin-1, also known as intelectin-1, is a novel adipokine produced mostly in visceral adipose tissue (85). Vaspin (visceral adipose tissue-derived serpin) is a member of serine protease inhibitor family first isolated by from visceral white adipose tissues of Otsuka Long-Evans Tokushima fatty rat (86). Although not often considered as an adipokine, adipose tissue releases free fatty acids to the systemic circulation, which contribute to the development of insulin resistance, pro-inflammatory, and pro-thrombotic state (87). In addition, obesity also leads to disturbances in the gut microbiota, which result in the leakage of lipopolysaccharide and other bacterial fragments to the systemic circulation, eventually leading to an inflammatory response (88, 89). Although the pathophysiological roles of these adipokines have been well studied in diabetic and obese patients, data on their relevance in the PD population are scarce.

## Insulin Resistance and Metabolic Syndrome

Another major mechanism that obesity leads to adverse clinical outcome is the concomitant insulin resistance and metabolic syndrome. Obesity is well reported to be associated with insulin resistance, and the major mechanism is *via* its effect on the incretin axis, and glucagon-like peptide 1 (GLP-1) is probably the most important mediator. In essence, GLP-1 is a 30-amino acid peptide hormone produced in the intestinal epithelial endocrine L-cells by differential processing of proglucagon, and is the major incretin hormone (90). One of the most important functions of GLP-1 is to, *via* its specific receptor, amply the post-prandial insulin secretion (91). The activity of GLP-1 is regulated by its degrading enzyme dipeptidyl peptidase-4 (DPP-4), which is also known as the T-cell antigen CD26. DPP-4 is an integral membrane protein expressed on many cell types, and is also shed from the membrane and circulates as a soluble protein in the plasma (92). Under physiological conditions, GLP-1 is the major substrate of the circulating DPP-4 (92). In obese patients, the secretion of GLP-1 is reduced, while that of DPP-4 is increased (93, 94). The alteration in the incretin axis is the major contributing factor of insulin resistance, dyslipidemia, and atherosclerosis in obesity (95, 96). In addition to its metabolic effects, the incretin axis is also involved in inflammation (97). In monocytes and macrophages, GLP-1 agonists and DPP-4 inhibitors suppresses the action of protein kinase A, which leads to a reduction in inflammatory cell infiltration into the arterial wall (97). In addition, DPP-4 also has many substrates other than GLP-1 that are implicated in the generation of inflammation (98). Dedicated data on insulin resistance and the alterations of incretin axis in PD patients, however, are scarce.

First recognized in its extreme form as Reaven’s syndrome X (99), metabolic syndrome began as a loosely defined clustering of major risk factors for cardiovascular diseases and type 2 diabetes (100). With the improving case recognition and definition, obesity has become a key element of the constellation of metabolic syndrome, as emphasized by the revised criteria for metabolic syndrome by International Diabetes Federation (IDF) (100). In the general population, metabolic syndrome is strongly associated with overall mortality, cardiovascular disease, and stroke (101). It has been suggested that any adverse outcome associated with obesity is the result of metabolic syndrome rather than obesity per se (102, 103). However, the definition of metabolic syndrome and its prognostic implication in PD remain controversial (71, 104). Traditional criteria for metabolic syndrome is not applicable to PD patients because they tend to have a larger waist circumference, there is never a genuine “fasting” state, and the confounding effect of excessive body fluid is considerable. In a retrospective analysis of 329 prevalent PD patients, the agreement between four sets of diagnostic criteria for metabolic syndrome was at best fair to moderate (104). In this study, metabolic syndrome was present in 53.2%, 53.8%, 60.5%, and 66.3% of the PD patients according to the original World Health Organization (WHO) criteria, the International Diabetes Federation (IDF) criteria, the original National Cholesterol Education Program (NCEP) criteria, and the modified NCEP criteria, respectively (104). However, the overall survival, cardiovascular survival, or technique survival did not differ between patients with and without metabolic syndrome, irrespective to diabetic status and diagnostic criteria being used (104), indicating that unlike the general population, metabolic syndrome may not be an important prognostic indicator of PD patients.

## New-Onset Glucose Intolerance After PD

A major cause of weight gain in PD patients is glucose absorption from the PD solution, and an important confounding factor that the weight gain may lead to adverse clinical outcome is the development of new-onset diabetes after PD. Since glucose is the most commonly used osmotic agent in commercial PD solution, it has been estimated that PD patients derive about 20% of their total daily energy intake from the glucose in PD solutions, which corresponds to a daily energy intake of 4 to 13 kcal/kg body weight (105). Our previous study of 252 non-diabetic incident patients found that fasting plasma glucose was above 7.0 mmol/l in 27.3% non-diabetic incident PD patients (106). In this study, obese patients did not have a higher risk of new-onset diabetes after PD, but even mild fasting hyperglycemia (fasting plasma glucose >5.6 mmol/l) was associated with a worse survival (106). In contrast, Dong et al. (107) examined total of 612 non-diabetic PD patients, and found that new-onset diabetes after PD was present in only 32 patients (5.2%). In this study, high BMI and C-reactive protein levels are major predictive factors for the development of new-onset diabetes and impaired glucose tolerance in PD patients (107). In a recent analysis of 1681 incident PD patients, Than et al. (22) noted that the fasting plasma glucose level after initiation of PD in patients without pre-existing diabetes rose gradually from 5.9 ± 2.0 to 6.4 ± 2.2 mmol/l from 1995 to 2019, and the incidence of new-onset diabetes increased from 18.0% to 23.3% during this period (22). In this study, there was a modest but statistically significant correlation between post-dialysis fasting plasma glucose level and baseline BMI in non-diabetic patients; new-onset diabetes was present in 37.9% patients with baseline BMI 19.0 to 23.9 kg/m2, but 59.0% of those with baseline BMI above 25.0 kg/m2 (22). However, outcome data were not analyzed in this study, and it remains uncertain whether the new-onset diabetes after PD directly causes any adverse outcome, or the glucose load from PD simply unmasks the underlying occult metabolic problems that is associated with CVD.

## Fluid Overload as a confounding Factor

Another important factor that confounds the relation between obesity and clinical outcome is the effect of fluid overload. A patient with a large body weight may be the result of being obese or having fluid overload, and any weight gain in PD patients may be due to increase in adipose tissue mass or worsening of fluid overload. In an early case control study of predominantly CAPD patients, diabetes as well as noncompliance with fluid restriction, salt restriction, and performance of dialysis exchange were important predictive factors of symptomatic fluid overload (108). As expected, peripheral edema, pulmonary congestion, pleural effusions, and arterial hypertension were all common manifestations of symptomatic fluid overload in this study (108).

In the context of obesity assessment, asymptomatic fluid overload is also common in PD patients (109). In the Initiative for Patient Outcomes in Dialysis-Peritoneal Dialysis (IPOD-PD) study that examined 1092 patients from 135 centers in 32 countries, only 38.7% patients had normal hydration, while fluid overload of over 1.1 L was present in 56.5% of them, and the median fluid overload was 2.0 L for male patients (110). Notably, most of the patients were already overhydrated before the initiation of PD, including those without a documented diagnosis of congestive heart failure (110). In a study that recruited 122 asymptomatic prevalent Chinese PD patients, Kwan et al. (111) reported that fluid overload, defined as over-hydration volume ≥1 L, was present in 72.1% patients, while 20.5% patients had over-hydration ≥5 L. Another study that recruited 307 prevalent Chinese CAPD patients with a median duration of PD 14.6 months found that fluid overload, as defined by extracellular to total body water ratio ≥0.40, was present in 66.8% patients, and over half of them had no symptom related to fluid overload (112). Kim et al. (113) further reported from a study of 284 prevalent PD patients that 68% patients who had fluid overload at baseline remained persistently hypervolemic one year later, and nearly 20% of the entire cohort had chronic fluid overload.

Fluid overload is a well reported predictor of adverse outcome in PD patients. Guo et al. (112) found that the cardiac event rate after 1 year of follow-up was significantly higher in the patients with fluid overload than those with normal hydration (17% vs 7%). In a study of 529 patients, O’Lone et al. (114) noted that the overhydration indices (including the absolute volume of overhydration, and its ratio with extracellular water volume) were independent predictors of all-cause mortality. Fluid overload is directly linked to increased cardiovascular morbidity and mortality (114, 115). Even among asymptomatic PD patients, Ng et al. (29) found that the volume status independently predicted patient survival and cardiovascular events (29). In this study of 311 incident PD patients, each 0.1 unit increase in extracellular-to-intracellular volume ratio was associated with 24.5% decrease in overall survival and 18.7% decrease in cardiovascular event-free survival (29). Longitudinal study with repeated bioimpedance spectroscopy measurement further showed that PD patients who remained persistently hypervolemic had a 2.8-fold increase in risk of transferring to chronic hemodialysis (113).

Traditionally, fluid overload is considered as a result of underlying cardiac disease or inadequate dialysis. More recently, it is increasingly recognized that fluid overload per se directly contributes to the pathogenesis and progression of cardiovascular disease. Specifically, fluid overload causes dysfunction of the gut permeability barrier dysfunction, a phenomenon that is particularly prominent in the context of CKD (116). It has long been reported that plasma levels of endotoxin, the major bacterial cell fragment that is implicated in the generation of systemic inflammatory response and cardiovascular disease, were higher in edematous heart failure patients than in non-edematous patients and healthy volunteers (3). In CKD, small intestinal water content correlated with plasma endotoxin level, suggesting that bowel wall edema leads to gut permeability barrier dysfunction (117, 118), which is postulated to facilitate the translocation of bacterial fragments to the systemic circulation, leading to systemic inflammation and cardiovascular diseases.

## Obesity in Different Compartments

In the recent years, it is increasingly recognized that obesity is an over-simplified concept. In the general population, it has long been recognized that central obesity, i.e. the accumulation of fat around the mid-body section, increases the risk of glucose intolerance, dyslipidemia, cardiovascular disease, Alzheimer’s disease, and several types of cancer (119, 120). Patients with a waist-to-height ratio exceeding 0.5, despite a normal BMI, had elevated mortality risk for cardiovascular and metabolic disease (121). It has been proposed that the accumulation of fat in the body close to vital organs in the abdomen leads to a low-grade systemic inflammatory state (122, 123). In PD patients, the assessment of central obesity by waist circumference is theoretically confounded by the instillation of PD solution into the peritoneal cavity. Nonetheless, waist conference remains a reasonable marker of abdominal adiposity in PD patients. In a study of 107 prevalent PD patients, Kamimura et al. compared waist circumference measured at umbilicus level to formal trunk fat measurement by dual-energy x-ray absorptiometry, the agreement (as determined by the kappa statistic) between waist circumference and trunk fat was 0.59, and the change in waist circumference significantly correlated with changes in trunk fat after 6 months (r = 0.49), both indicate a moderate agreement between the methods (124).

However, the differentiation between central and peripheral obesity may not be sufficiently accurate because adipose tissue in human body could be divided into several distinct compartments (125). Traditionally, adipose tissue is classified by morphology into white, brown, or beige subsets (126). Although brown and beige adipose tissue contribute little, if any, to the total body adipose tissue mass in normal adults (126), white adipose tissue per se could be further classified by its location into subcutaneous and visceral ones, which constitutes 80% and 20%, respectively, of the white adipose tissue mass (127). The usual concept of central obesity, therefore, encompasses visceral adipose tissue (located intra-abdominally, in the omentum, and adjacent to internal organs) as well as subcutaneous adipose tissue in the abdominal wall (127). There is, however, emerging evidence that subcutaneous and visceral adipose tissues are different in their metabolic profile and clinical implications. In essence, current data suggest that visceral adipose tissue is the key determinant of insulin resistance, metabolic disturbance, and probably adverse cardiovascular outcome in obesity, while subcutaneous adipose tissue appears to have a small protective effect (125, 128–130).

It is important to note that the differential effect and prognostic implications of visceral and subcutaneous fat have not been explored in the PD population, but in an observational study of 115 PD patients, Bazanelli et al. (124) examined the change in body fat distribution over time by dual-energy x-ray absorptiometry. In this study, the overall BMI gradually increased with the vintage of PD, but there was a gradual decline in the amount of truncal fat simultaneously (124). The mechanism and prognostic implication of the progressive change in body fat distribution in PD patients require further studies.

## Change in Body Composition After PD

As discussed above, a large body weight may be the result of excessive body water rather than a higher adipose tissue or muscle mass. Similarly, progressive weight gain after PD may be caused by fluid overload rather than obesity. Nonetheless, early reports showed that the content of body fat, especially intra-abdominal fat, increases after PD treatment (131–133), while the magnitude of overhydration did not appear to increase during the first two years of PD (134). In a prospective study of 19 PD patients, Fernström et al. (131) measured the body fat by serial computed tomography (CT) and dual energy x-ray absorptiometry (DEXA) and found that although the overall body weight increased insignificantly from 67.1 to 68.4 kg over an average of 7 months on PD, the intra-abdominal fat area increased by 22.8%, and the percentage of total body fat content increased from 27.8% to 30.9%. In a study with an extended follow up of 79 months, Søreide et al. (132) examined the percentage of total body fat of 8 PD patients by means of a computerized model of near infrared interactance and found that the total body fat content increased from 19.8% to 22.5%, while the body weight had no change. When individual adipose tissue compartments were monitored over 12 months in 60 Korean PD patients by bioelectric impedance analysis and computed tomogram, Choi et al. (133) found that their body weight continued to increase in during the first 12 months on PD, but visceral and subcutaneous fat mass increased only during the first 6 months, and then decreased from 6 to 12 months. In this study, patients with more visceral fat mass at the start of PD had less gain of visceral fat mass during the first 6 months, and those with more subcutaneous fat mass at the start of PD had less gain of subcutaneous fat mass, indicating that regression-to-mean is an important confounding factor (133). More importantly, the change in body weight was not associated with the change in visceral or subcutaneous fat, suggesting that hydration status was the major determinant of body weight change (133).

The best data in this aspect came from the Initiative for Patient Outcomes in Dialysis-Peritoneal Dialysis (IPOD-PD) study (135), which recruited 1054 incident PD patients in 135 centers from 28 countries, and their volume status was measured by bioimpedance spectroscopy before the start of PD and then every 3 months for 3 years. In this study, the mean volume overload was 1.9 L before the initiation of PD, which was reduced to 1.2 L after one year of PD, and it remained stable at year 2 and 3 (135). Baseline clinical parameters and PD prescription did not predict the change in volume status over first 6 months in this study, but a relative volume overload over 17.3% was independently associated with a higher risk of death (adjusted hazard ratio 1.59) (135). In a prospective study of 155 incident PD patients with a median follow-up of 12 months, Jaques et al. (136) noted that although fluid overload is common, volume status could be reasonably controlled when the PD prescription was tailored to patient’s individual characteristics, while the level of residual renal function, modality of PD (CAPD versus machine-assisted PD), and peritoneal characteristics are not decisive in this matter.

Although fluid status may be an important confounding factor, there is emerging evidence that the progressive accumulation of fat in PD does have clinical impact, even though it may be “masked” by the concomitant changes in other body composition. In an observational study of 160 PD patients followed for 2 years, Kim et al. (137) showed that although the body weight appeared to be static, loss of lean tissue mass (i.e. muscle mass) and gain in adipose tissue mass were observed after 2 years of PD in over 30.5% and 44.3% of patients, respectively. However, the impact of obesity in sarcopenic patients has not been well-studied. The loss of lean tissue and gain in adipose tissue mass were both reported to be independent risk factors for all-cause mortality after adjusting for demographic, biochemical, and cardiovascular parameters (137). In the IPOD-PD patient cohort, the use of hypertonic and glucose solutions were significantly associated with a decrease in lean tissue mass and an increase in adipose tissue mass over time (138). In this analysis, lean tissue mass inversely correlated with the risk of death, while a high adipose tissue mass (as represented by the fat tissue index) was associated with a higher sub-distributional hazard ratio for the risk of death when compared with the median as a reference (138).

## Conclusion

**Figure 1:**
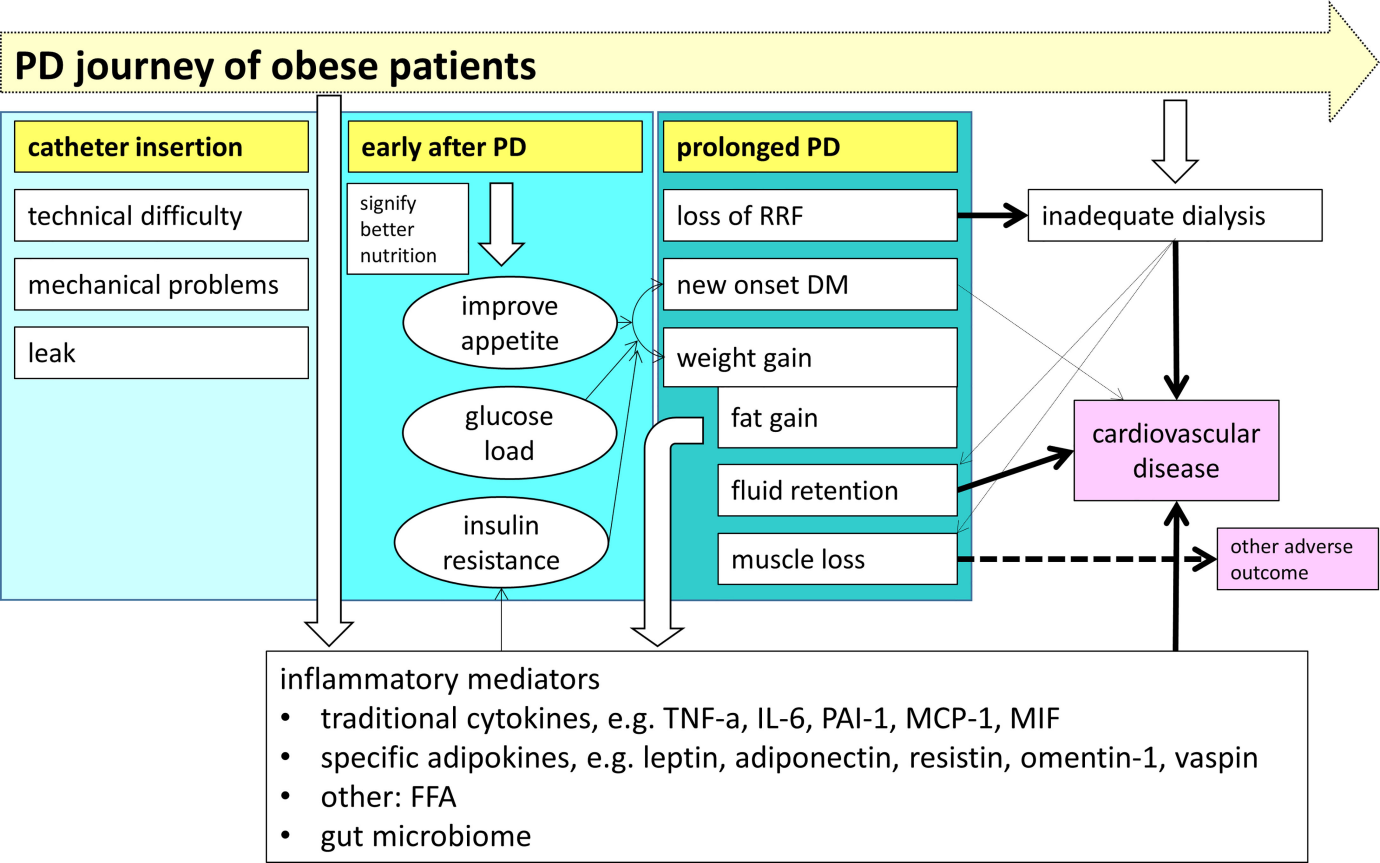
The impact of obesity on the clinical outcome along the journey of patients treated with peritoneal dialysis (PD). Before PD is started, obesity is associated with several specific problems during dialysis catheter insertion. During the early phase of PD, obesity is a surrogate marker of a better nutritional status. With time on PD, there is a gradual decline in skeletal muscle mass and an increased in body fat and fluid, resulting in a progressive overall weight gain. Obesity directly contributes to the systemic inflammatory state *via* a panel of inflammatory mediators, and also aggravates the problem of inadequate dialysis when the patient become anuric, both lead to the adverse outcome of chronic PD patients. (PD, peritoneal dialysis; RRF, residual renal function; DM, diabetes mellitus; TNF-α, tumor necrosis factor alpha; IL-6, interleukin-6; PAI-1, plasminogen activator inhibitor type 1; MCP-1, monocyte chemoattractant protein-1; MIF, macrophage migration inhibitory factor; FFA, free fatty acids).

The above discussion indicates that the relationship between obesity and the outcome of PD patients is complicated, and body weight is not a reliable measurement for obesity. In essence, obesity has variable clinical effects along the journey of PD patients, as summarized in **Figure 1**. Obesity is associated with various complications during PD catheter insertion. At the initiation of PD, obesity is probably a marker of better nutrition, which explains its association with improved clinical outcome in some observational studies. Nonetheless, adipose tissue secretes a number of inflammatory mediators, and obesity aggravates the problem of inadequate dialysis in anuric patients. With time on PD, there is usually weight gain due to increase in body fat and fluid accumulation, each of them independently leads to adverse outcome, especially an increase in cardiovascular risk. The concomitant decline in skeletal muscle mass may be masked, but will also contribute to the adverse clinical outcome.

## Strategies of Weight Loss

In obese patients with type 2 diabetes, weight loss is known to reverse the underlying metabolic abnormalities and improve glycemic control (139). In this group of patients, weight loss of 15% has a disease-modifying effect that could not be achieved by insulin or oral hypoglycemic agents (139). Furthermore, weight loss improves risk factors for cardio-metabolic disease and quality of life in this population (139). Given the complex relationship between obesity and the clinical outcome of PD, it remains to be determined whether weight loss confers an equivalent clinical benefit in obese PD patients.

If weight loss is considered desirable, dietary restriction and exercise is difficult to achieve and sustain the target body weight (140). In obese adults with normal kidney function, phentermine-topiramate and glucagon-like peptide-1 (GLP-1) receptor agonists, especially semaglutide, are the most effective drugs in reducing weight (141). Neither phentermine-topiramate nor GLP-1 receptor agonists, however, has been tested in obese PD patients. Given the accumulating evidence that GLP-1 receptor agonists reduce the cardiovascular events in patients with moderate to severe CKD (142), it would be important to explore the efficacy and benefit of using GLP-1 receptor agonists for obese PD patients. Surgical options, such as gastric bypass and sleeve gastrectomy, are highly effective in body weight control and have well documented metabolic benefits (143–145), but conversion to long term hemodialysis is necessary. Adjustable intragastric balloon is a minimally-invasive alternative that is feasible for the PD population but further studies are required.

## Gaps of Knowledge and Further Research Directions

The above discussion reviewed a wealth of literature on the complex relation between obesity and the prognosis of PD patients. Suffice to say, the impact of adipose tissue mass on the clinical outcome is not linear, is different from the relation observed in the general population, includes the risk of cardiovascular disease as well as that of conversion to hemodialysis, and there is a considerable confounding effect from the concomitant changes in other aspects of body composition (notably decline in muscle mass and fluid overload). There are many questions to be answered in this field, and we outlined in **Table 2** some of them which we considered particularly interesting. Notably, recent data showed that glucagon-like peptide-1 (GLP-1) receptor agonists, such as semaglutide, as well as dual gastric inhibitory peptides (GIP) and GLP-1 receptor agonist, such as tirzepatide, are effective treatment of obesity and probably reduce the risk of cardiovascular events in obese or diabetic patients (111, 146–148). It would be interesting to explore whether these agents are equally beneficial in PD patients.

**Table 2:** Gaps of knowledge and further research directions.

◼	What are the adipocyte dysfunctions in CKD and PD patients?				
◼	Differentiate the functions and abnormalities between adipocyte and stromal cells in the adipose tissue.				
◼	How does acute events (notably peritonitis) affect body fat content of PD patients?				
◼	What are the prognostic impact of different body components (i.e. fat content, muscle mass, and fluid overload)?				
◼	How to measure specific compartments of adipose tissue (especially visceral fat content)?				
◼	What is the optimal treatment of new onset diabetes after PD? What should be the target of glycemic control?				
◼	How to preserve muscle mass and avoid excessive fat accumulation at the same time?				
◼	What is the role of non-pharmacological therapy (e.g. exercise training)?				
◼	Is there any role of the novel anti-obesity agents (e.g. glucagon-like peptide-1 receptor agonists) in PD patients?				

## Author Contributions

JN and WT were responsible for literature review and writing the first draft of the manuscript. CS was responsible for the overall idea, organization and revision of the manuscript. All authors contributed to the article and approved the submitted version.

## Funding

This study was supported by the Richard Yu Chinese University of Hong Kong (CUHK) PD Research Fund, and CUHK research accounts 6905134 and 7105912. The funders had no role in study design, data collection and analysis, decision to publish, or preparation of the manuscript.

## Conflict of Interest

The authors declare that the research was conducted in the absence of any commercial or financial relationships that could be construed as a potential conflict of interest.

## Publisher’s Note

All claims expressed in this article are solely those of the authors and do not necessarily represent those of their affiliated organizations, or those of the publisher, the editors and the reviewers. Any product that may be evaluated in this article, or claim that may be made by its manufacturer, is not guaranteed or endorsed by the publisher.
